# Burnout Status of Italian Healthcare Workers during the First COVID-19 Pandemic Peak Period

**DOI:** 10.3390/healthcare9050510

**Published:** 2021-04-28

**Authors:** Chiara Conti, Lilybeth Fontanesi, Roberta Lanzara, Ilenia Rosa, Robert L. Doyle, Piero Porcelli

**Affiliations:** 1 Department of Psychological, Health, and Territorial Sciences, University “G. d’Annunzio” of Chieti-Pescara, 66100 Chieti, Italy; lilybeth.fontanesi@unich.it (L.F.); piero.porcelli@unich.it (P.P.); 2 Department of Dynamic and Clinical Psychology, “Sapienza” University of Rome, 00185 Rome, Italy; roberta.lanzara@uniroma1.it (R.L.); ilenia.rosa@uniroma1.it (I.R.); 3 Department of Psychiatry, Harvard Medical School, Boston, MA 02115, USA; rldoyle1@bics.bwh.harvard.edu

**Keywords:** burnout, COVID-19, healthcare workers, psychological well-being, post-traumatic symptoms, psychological distress

## Abstract

The pandemic of 2019 coronavirus disease (COVID-19) has burdened extraordinary psychological stress on the healthcare workforce. The present survey aimed to examine the personal resources and psychological symptoms associated with burnout in 933 healthcare workers in Italy during the COVID-19 outbreak period. Sociodemographic and occupational data, depression, anxiety, burnout, and post-traumatic symptoms, as well as psychological well-being, were cross-sectional assessed through an online questionnaire. A considerable part of the sample scored over the clinical levels of depression (57.9%), anxiety (65.2%), post-traumatic symptoms (55%), and burnout (25.61%). Working in the front-line (*p* < 0.05), being part of the medical staff (*p* < 0.05), experiencing lower levels of psychological well-being (*p* < 0.001), and higher levels of post-traumatic symptoms (*p* < 0.001) independently explained 38% of burnout variance. The healthcare industry, services, and professionals should be aware of the harmful effects of COVID-19 on healthcare workers and take adequate preventive measures.

## 1. Introduction

The rapid global spread of the Coronavirus Disease 2019 (COVID-19), first identified in Wuhan, China, and now spread worldwide [1], has rapidly raised concerns about the well-being of healthcare workers (HCWs) [2]. In Italy, the total number of cases went beyond 2,739,591 people, and the number of people killed by COVID-19 is about 94,171 [3]. About 110,647 HCWs have been infected, and 244 have died during the epidemic in Italy [4] due to the proximity with infected patients. Since the inception of the pandemic spread in December 2019, each hospital department had worked in emergency mode and has been on daily alert to prevent a possible aggravating of the situation. Much of the medical staff were required to work in unfamiliar wards or were on standby to be called to the hospital from their home offices [5]. In the second phase of the pandemic, all HCWs continue to cope with massive adjustments at work. Management of the crisis has been associated with stress reactions that occurred on both the physical and mental levels. Particularly critical is the work in close contact with patients positive for COVID-19 or with patients who are in the hospital with suspected COVID-19 infection [6,7]. Studies conducted in China reported that HCWs with a higher burden of workload and treating patients with life-threatening medical conditions are experiencing severe psychological pressure, even psychopathological conditions [8,9]. Compared with non-medical healthcare staff (NMHS), medical healthcare staff (MHS, i.e., physicians and nurses) had more psychosocial problems during the COVID-19 outbreak [8,10]. Studies showed that front-line MHS reports high levels of stress that result in depression, anxiety, somatic symptoms, and post-traumatic stress disorder (PTSD) due to the clinical work and close contact with COVID-19 patients during the outbreak [11,12]. A meta-analysis on HCW reported that the most common indicators of psychological impact across COVID-19 studies are anxiety and depression, with an approximate prevalence of 33% and 28%, respectively [2].

The overall psychological distress in HCWs has been linked to burnout, a work-related professional hazard acquired while providing healthcare for patients. Burnout syndrome can include symptoms of emotional exhaustion, depersonalization, cognitive weariness, physical fatigue, and disengagement [13]. Burnout can lead to physical and mental health problems and may also affect the healthcare quality for patients [14,15,16]. The negative consequences of HCWs’ burnout include inadequate clinical care, increased medical errors, patient dissatisfaction, dysfunctional relations with colleagues, the contagion of burnout, substance abuse or self-medication, depression, and even suicide [17]. In HCWs, the risk of burnout is higher in younger individuals, women, those who work long hours, and permanently employed professionals [18,19,20]. Stress management programs that range from relaxation to psychological support have been found to be of utmost significance in preventing and treating burnout [21]. In Italy, burnout among healthcare workers, mainly medical staff, is becoming an occupational hazard, with its rate reaching between 27% and 59% in some clinical specialties [22,23]. Morgantini and colleagues [24] identified four major occupational risk factors for burnout in HCWs during the COVID-19 outbreak, including limited organizational support, high levels of workload, job stress, and time pressure. An Italian study [25] examined burnout in HCWs during the COVID-19 epidemic and reported a significant main effect of sex and occupational role on burnout symptoms among front-line HCWs. A recent review [26] showed that novel COVID-19 has heightened existing challenges that HCWs face, such as increased workload, which is directly correlated with increased burnout. However, front-line healthcare work during the COVID-19 pandemic does not necessarily correlate with increased burnout and is an area for more research. Both job demands and personal resources are thought to affect HCWs’ psychological health, including the experience of burnout [27]. Given the countless difficulties that HCWs face in their clinical work, especially in periods of crisis, and the intense exposure to general psychological distress, it is essential for the well-being of HCWs to strengthen the use of their personal resources and coping strategies and to use them actively especially in times of crisis. The number and type of resources are among major factors in evaluating whether people may come out of a crisis as more weakened or more strengthened. According to the six-factor model of psychological well-being (PWB) developed by Carol Ryff [28], personal resources include positive relationships with others, personal mastery, autonomy, a feeling of purpose and meaning in life, and personal growth and development. Stress- and health-related aspects as lower levels of salivary cortisol, lower cardiovascular risk, and lower inflammation response to psychological stress were observed in people with higher levels of PWB [29,30,31]. PWB is also related to optimal sleep patterns and lower levels of depressive and anxiety symptoms [32]. Moreover, adults with high levels of PWB and limited psychological distress had fewer chronic medical conditions [33] and showed lower healthcare use and greater workplace productivity [34] than people with lower levels of PWB. PWB may therefore buffer the development of negative outcomes, including medical errors, across diverse work contexts [35]. For instance, a sense of meaning, hope, optimism, and resilience seem to buffer the effects of workload on burnout in HCWs [36,37,38,39].

We conducted an online survey to examine the psychological risk and protective factors of burnout in Italian HCWs during the first COVID-19 outbreak period between March and May 2020. In particular, the aim of the study is three-fold: (a) to investigate whether HCWs with burnout have higher workload and psychological distress symptoms (i.e., depression, anxiety, post-traumatic), and lower personal resources than those without burnout; and (b) to examine whether and to what extent sociodemographic characteristics, workload, personal resources, and psychological distress symptoms are associated with burnout. Based on previous literature, we expected that: (a) HCWs with burnout would exhibit more features of increased workload, psychological distress symptoms, and lower personal resources than those without burnout; and (b) sociodemographic characteristics, higher workload, higher psychological distress symptoms, and lower personal resources would be the predictors of burnout.

## 2. Materials and Methods

### 2.1. Participants and Procedure

A sample of 1185 HCWs who were working during the COVID-19 epidemic was recruited through an online survey. The research follows a cross-sectional design. Data collection occurred from 30 March to 3 May 2020, concurrently with the peak of the COVID-19 outbreak when strict lockdown measures for all people in Italy were underway. HWCs were invited to participate in the online survey through the Qualtrics platform (https://www.qualtrics.com). Participants were recruited through social network communities using snowball sampling. To optimize ecological validity, all HCWs from 18 to 65 years old were included since this range represents the minimum and maximum ages to be legally employed in the National Health Service (NHS) in Italy. Being at least 18 years old and working in the NHS were considered inclusion criteria. Only participants who satisfied the inclusion criteria and completed the survey were included in the analysis, with the final sample composed of 933 subjects (78.73%). The sample included 66.35% MHS (24% physicians, 42.3% nurses) and 33.65% NMHS (10.6% technicians from radiology and laboratory medicine, 17.7% unlicensed assistive personnel, 5.4% other hospital staff as pharmacists or ambulance drivers) from all Italian regions. These percentages are representative of the proportion of workers in the different categories of the Italian NHS [40]. Included participants were mostly females (76.5%) and aged 41.77 ± 12.08 years old (median = 41.00). All participants provided online informed consent to participate. The study was designed and carried out in accordance with the World Medical Association Declaration of Helsinki and its subsequent revisions [41] and approved by the Ethics Committee of the Department of Psychological, Health and Territorial Sciences (DiSPuTer) of University G. d’Annunzio—Chieti-Pescara.

### 2.2. Measures

#### 2.2.1. Sociodemographic and Occupational Characteristics

Sociodemographic and occupational data were self-reported by the participants, including sex, age, educational level, workload characteristics (i.e., working position, patients’ death), and different healthcare professions (MHS and NMHS). Working position was assessed as being directly involved (front-line) or not (second-line) in the clinical management of patients with suspected or confirmed COVID-19; and experiencing a patients’ death, which was defined as at least one of the subject’s own patients who died from COVID-19.

#### 2.2.2. Burnout

The Stanford Professional Fulfillment Index (PFI) is a 16-item outcome measure that assesses both burnout (work exhaustion (WE) subscale and interpersonal disengagement (ID) subscale) and professional satisfaction (professional fulfillment (PF) subscale) in physicians during the past two weeks [42]. Response options are on a 5-point Likert scale ranging from 0 to 4 (“not at all true” to “completely true” for PF items and “not at all” to “extremely” for WE and ID items). Scale scores are calculated by averaging the items scores of all items within each corresponding scale, such that all subscale scores also range from 0 to 4. Scoring > 1.33 is considered the threshold for clinical levels of burnout. Within this sample, Cronbach’s α was 0.84 for the total scale, 0.75 for the PFI-WE, 0.84 for the PFI-ID, and 0.74 for the PFI-PF subscales.

#### 2.2.3. Depression Symptoms

The self-report 9-item patient health questionnaire (PHQ-9) was used to measure depression symptoms [43]. The PHQ-9 is widely used in primary care and other clinical and research settings for screening depression. Participants were asked to report the severity of each symptom during the last two weeks on a 4-point Likert scale from 0 (“not at all”) to 3 (“nearly every day”). The total PHQ-9 scores range from 0 to 27, scores of <5 represent the absence of depression symptoms, and higher scores indicating greater severity of depression. The PHQ-9 has been shown to be provided with sound psychometric characteristics [44]. Within this sample, Cronbach’s α was 0.88.

#### 2.2.4. Anxiety Symptoms

The self-report 7-item generalized anxiety disorder scale (GAD-7) was used to assess anxiety symptoms. The GAD-7 is a self-report measure that is widely used in clinical and research settings for screening anxiety [45]. Subjects were asked to rate how often they have been bothered by each symptom during the past two weeks. Responses are scored on a 4-point rating scale from 0 (“not at all”) to 3 (“every day”). Total scores range from 0 to 21, scores of <5 represent the absence of anxiety symptoms, and higher scores reflecting higher severity levels of generalized anxiety disorder symptomology. The GAD-7 has good reliability, construct, factorial, and procedural validity [46,47]. Within this sample, Cronbach’s α was 0.91.

#### 2.2.5. Post-Traumatic Stress Disorder Symptoms

The 22-item impact of event scale, revised (IES-R) Italian version [48] was used to evaluate the psychological impact of the COVID-19 outbreak. Respondents were asked to complete the questionnaire regarding symptoms in relation to the concerns the last week and to refer to the pandemic-related experience. The IES-R is a widely used measure of psychologically distressing symptoms due to a specific stressful event [49]. The scale produces three subscale scores for assessing intrusive thoughts (IT), hyperarousal (H), and avoidance symptoms (A) in the previous 7 days with respect to the event. Items are rated on a 5-point Likert scale ranging from 0 (“not at all”) to 4 (“extremely”). Total score ranges from 0 to 88, with scores of 33 or higher reflect probable PTSD [50]. Within this sample, Cronbach’s α was 0.92 for the total scale, 0.85 for the IES-IT, 0.80 for the IES-H, and 0.76 for the IES-A subscales.

#### 2.2.6. Psychological Well-Being

Psychological well-being was assessed using the self-report 18-item psychological well-being scales, revised (PWB-R) [51]. Responses are scored on a 7-point Likert scale from 1 (“strongly disagree”) to 7 (“strongly agree”). The PWB-R total scores ranging from 18 to 126, with higher scores indicate higher levels of psychological well-being. The questionnaire produces six subscales: (1) autonomy, (2) environmental mastery, (3) personal growth, (4) positive relations with others, (5) purpose in life, and (6) self-acceptance. Reliability and validity have been demonstrated. Constant with Italian validation [52], within our sample, Cronbach’s α was 0.77 for the total scale and ranges from 0.49 to 0.66 for the individual scales.

### 2.3. Statistical Analysis

Between-group differences for burnout (PFI ≥ 3) in sociodemographic, occupational, and psychological variables were performed using Student’s *t*-test or chi-square test (χ^2^). The effect size was measured using the standardized mean difference (Cohen’s d). A Cohen’s d of 0.20–0.50, 0.50–0.80, and >0.80 correspond to small, medium, and large effects [53]. Eta-square (η²) was used to evaluate the effects size between subjects- Eta-squares values ranging 0.02–0.12 represent the presence of small effect size, 0.13–0.25 a moderate effect size, and >0.26 a large effect size [54]. Pearson’s correlation coefficient was used for the associations between burnout and sociodemographic, occupational, and psychological variables.

Multivariate hierarchical regression analysis was performed to identify major factors that best predict burnout. Burnout was considered as a dependent variable, and sociodemographic, occupational, and psychological variables were considered independent variables. Four regression models were estimated, and regression coefficients and the corresponding *p* values were calculated. In the first step, the sociodemographic characteristics (age, sex) were entered. To evaluate the contribution of COVID-19 associated factors to burnout before adjusting for psychological variables, working position, patients’ death, and different healthcare professions were included in the second model. To estimate separately the influence of personal resources and psychological symptoms, psychological well-being was added in the third step; anxiety, depression, and post-traumatic symptoms were added in the fourth step. The multicollinearity analysis, performed to evaluate the degree of intercorrelation among the estimated variables, indicates the independent role of any specific variables. There were no missing data due to the forced response mode available on Qualtrics. All statistical analyses were computed using IBM SPSS Statistics version 25 (IBM Corp., Armonk, NY, USA) [55].

## 3. Results

Table 1 reports the sociodemographic, workload characteristics, and psychological symptoms of the total sample and healthcare professions subgroups. Sex was not equally represented in the sample, and women were significantly more prevalent than men in all professional categories (χ^2^ = 35.51, *p* < 0.001, η^2^ = 0.19). To a closer look, standardized deviates indicate that more men and fewer women were represented within physicians when compared to other healthcare profession subgroups. Front-line workers were more prevalent in the sample (76%). Other hospital staff workers and UAP, as expected, were more present in second-line healthcare units than other professionals (χ^2^ = 27.37, *p* < 0.001, η^2^ = 0.17). The number of HCWs who experienced at least one of their patients who died from COVID-19 (40.6%) was lower than who did not experience that. As also expected, other hospital staff members experienced even lower patient deaths compared to other categories (i.e., physicians, nurses, technicians, and UAP) (χ^2^ = 21.48, *p* < 0.001, η^2^ = 0.15). A considerable proportion of participants scored over the clinical cut-off of PHQ-9 (57.9%), GAD-7 (65.2%), IES-R (55%), and PFI-tot (25.61%), but no significant differences were found between the HCWs subgroups, except for other hospital staff that scored over the clinical cut-off of PHQ-9 to a lesser extent than other HCWs subgroups (χ^2^ = 13.67, *p* = 0.008, η^2^ = 12).

Table 2 shows psychological symptoms, sociodemographic and occupational characteristics in the total sample and in the two groups categorized by clinically significant levels of burnout (PFI-tot ≥ 1.33). A slight majority of HCWs presenting burnout symptoms were younger (d = 0.20), working in the MHS (71.5%; η^2^ = 0.06), in the front-line (85.4%; η^2^ = 0.12), and had lost one of their patients due to COVID-19 (52.3%; η^2^ = 0.14). Burnout was associated with a much larger effect size with psychological variables. Participants with burnout had significant higher levels of depression (PHQ-9, d = 0.78), anxiety (GAD-7, d = 0.80), and post-traumatic symptomatology (IES-R, d = 1.03) as well intrusive thoughts (IES-IT, d = 0.81), hyperarousal (IES-H, d = 1.02), and avoidance (IES-A, d = 0.93), than participants without burnout. Moreover, HCWs with burnout showed lower levels of psychological well-being (PWB-R, d = 0.54), consistently in all PWB subscales but one (PWB-A), as reported by effects size ranging from d = 0.26 to d = 0.49.

Table 3 shows Pearson correlations between burnout scale total score, professional fulfillment, work exhaustion, and interpersonal disengagement, and the studied psychological variables, as well sociodemographic and occupational factors. The main results, according to the point-biserial correlation coefficients, show that the overall burnout score (PFI-tot) was highly positively associated with anxiety, depression, and post-traumatic stress symptoms with correlation values ranging from 0.47 to 0.57. In particular, work exhaustion (PFI-WE) had a large association with anxiety (GAD-7, r = 0.58), depression (PHQ-9, r = 0.52), and post-traumatic stress symptoms (IES-R scores ranging from r = 0.45 to r = 0.63), whereas professional fulfillment (PFI-PF) and interpersonal disengagement (PFI-ID) showed small to moderate correlation with PHQ-9, GAD-7, and IES-R (r values between 0.23 and 0.35). A low to small unilinear correlation was found between burnout scores, sociodemographic factors, work-related factors, and psychological well-being.

Table 4 shows four hierarchical regression models with a PFI-tot score as a dependent variable. As expected from unilinear regression (Table 4), the first two steps using sociodemographic and work-related variables explained a small portion of 2% of the PFI variance. Adding psychological well-being (β = −0.14, *p* < 0.001) in step 3 significantly explained an additional 9% of burnout variance that was further increased by a larger portion of explained variance of 22% when depression, anxiety, and post-traumatic stress symptoms were included as predictors in step 4, with only post-traumatic symptoms (β = 0.43, *p* < 0.001) showing the significant effect. The final model explained PFI variance by 38%.

## 4. Discussion

Italy was one of the most affected countries to deal with the new COVID-19 in the first period of the pandemic, during which the healthcare industry, services, and professionals had been forced to reconsider priorities and face new challenging situations. Since the beginning of the pandemic, the medical workforce took pains to struggle with the epidemic in the front-line and to protect public health. MHS worldwide, as the backbone of the fight in the first line of epidemic prevention and control, experienced significantly increased workload, high risk of infection, and work pressure [5]. These specific situations posed considerable stress on MHS, which led to severe psychological pressure, even depression, anxiety, insomnia, and distress [2]. The present study raises another important problem. The psychological pressure of HCWs can be a risk to that individual and the patients under his or her care. HCWs who are suffering from burnout syndrome have been shown to feel less involved in their relationship with patients [56], to make more medical mistakes [57], and even to compromise the clinical outcomes of patients [58,59]. The impact of the COVID-19 epidemic might be, therefore, greater and on a larger scale than a single individual psychological health level, affecting the quality of healthcare relationships and resulting in increased socioeconomic costs [57,60], thus reinforcing the need for psychological support for HCWs in their daily work.

The present study aimed to examine the sociodemographic characteristics, workload, personal resources, and psychological symptoms associated with burnout in HCWs in Italy during the COVID-19 outbreak. We investigated 933 HCWs from different regions in Italy. Up to 25% of the HCWs within our sample experienced clinically significant levels of burnout in their work during the pandemic, more than 50% depression symptoms, more than 60% anxiety symptoms, and more than 50% post-traumatic stress symptoms. Overall, more than half of the Italian HCWs participants experienced high levels of psychological distress. Consistent with previous literature, our findings suggest that COVID-19 represents an overwhelming situation affecting the general psychological health of HCWs [2].

In our first hypothesis, we expected that HCWs with burnout would show more features of workload, psychological distress symptoms, and lower personal resources than those without burnout. This hypothesis was fully confirmed. In line with previous studies [25,61], we found that participants with burnout were younger and MHS compared to older professionals who were not personally involved in medical work (NMHS). Moreover, our data revealed that participants who had alarming levels of burnout have worked more in the front-line, experienced the loss of more patients, and reported less personal resources and more depression, anxiety, and post-traumatic symptoms than those with a low level of burnout. Thus, suggesting that burnout was associated with the kind of professional role (being part of MHS) and clinical practice (working in front-line). This finding confirms previous research that close contact with patients may be an important driver of burnout [62]. This association might also be explained by the many clinical responsibilities that the HCWs had to face as well as the initial lack of safety at work, such as the insufficient understanding of the virus, the lack of prevention and control knowledge, the long-term workload, the high risk of exposure to patients with COVID-19, the shortage of medical protective equipment [63], the lack of sleep, and the exposure to critical life events [64].

In our second hypothesis, we expected that sociodemographic characteristics, higher workload, higher psychological distress symptoms, and lower personal resources would be the predictors of burnout. Consistently, we found that COVID-19-related variables such as working in the front-line directly with infected patients, as a part of the MHS, as well as low personal resources, and post-traumatic symptomatology, independently predicted the severity of burnout in our sample by explaining 38% of its variance. Studies on the role of traumatic events in HCWs have confirmed that post-traumatic distress has a wide influence on the onset of burnout symptomatology, as seen after natural disasters such as earthquakes and hurricanes in Italy and Japan [65,66,67]. Natural disasters, as well as the COVID-19 pandemic, are unexpected traumatic events that are likely to affect directly the personal life of HCWs. Recent researches on HCWs suggest that exposure to specific stressors, such as working with people who have experienced traumatic events, contributes to the development of burnout symptoms [68,69]. Professionals in contact with traumatized patients are exposed to trauma because of their direct experience with the event and the pressure they may feel in responding to traumatized patients’ needs [70,71]. In sum, the unexpected characteristics of the COVID-19 outbreak may have had prolonged effects for HCWs as either direct and indirect traumatic experiences, thus paving the way to clinically significant manifestations of burnout syndrome, particularly in MHS workers. Our results pointed out the influence of psychological well-being on burnout manifestations. A number of early studies suggest that in healthcare, a focus on personal resources is particularly important. For instance, a sense of meaning and a sense of well-being despite adversity (e.g., hope, optimism, resilience) seem to buffer the effects of workload on burnout in HCWs [36,37,38,39]. Studies suggest that many personal resources such as positive relationships with others, personal mastery, autonomy, a feeling of purpose and meaning in life, and personal growth and development, including in the broader construct of psychological well-being, can facilitate resilience to traumatic stressors by serving as a buffer against resource loss [28,72]. Indeed, individuals in stressful situations may feel as if they are losing their internal resources; this appears to be a risk factor for burnout [73]. There is evidence that increased resilience can be promoted by specific interventions leading to a positive evaluation of one’s self, a sense of continued growth and development, the belief that life is purposeful and meaningful, the possession of quality relations with others, the capacity to manage effectively one’s life and a sense of self-determination. For example, a decreased vulnerability to depression, mood swings, and anxiety has been demonstrated in high-risk populations after well-being therapy (WBT), based on Ryff’s conceptual model [74]. Literature shows that interventions designed to improve resilience in HCWs have efficacy in decreasing burnout, improving quality of life, and promoting healthy behaviors [75,76]. Taken together, our results show that post-traumatic symptoms may predispose HCWs to experience high burnout, and psychological well-being may protect HCWs from developing burnout.

Some limitations are to be acknowledged. First, our sample of HCWs included an unbalanced proportion between females and males. Women were over-represented in the total sample. Although this prevalence can bias our results, it should be noted that actually, it is a reliable picture of the sex-biased prevalence in healthcare professions in Italy [77]. Nonetheless, our data should be interpreted with caution because they may be related more closely to women rather than HCWs in general. In the same vein, more than three-quarters of responders worked in front-line, and the generalizability of our findings might be limited to HCWs working in more stressful conditions. Second, the cross-sectional nature of our data does not allow one to establish the direction of causality, and longitudinal studies are needed to evaluate whether the current stressful condition will be persistent and the associated consequences it may have on the individual characteristics and the burnout of HCWs. For this purpose, a follow-up online survey has been planned by our research group. Third, all the questionnaires we used have been validated and have shown excellent psychometric properties in the usual administration. However, research has shown that self-completed questionnaires, electronic, and online administration can be used interchangeably for research [78,79], thus suggesting that the psychometric features of scales can be reliably considered. Fourth, in our study, estimates of PWB-R internal consistency (Cronbach’s α) coefficients were low to modest, ranging from 0.49 to 0.66. The modest α coefficients likely reflect the small number of indicators per scale and the fact that the authors of this scale [51] chose items to represent the conceptual breadth within each construct rather than to maximize internal consistency. Fifth, the online administration of a survey is subject to responder bias. Particularly with HCWs during the dramatic pandemic, people who agreed to answer questions on their psychological health might be much more motivated to participate if psychologically distressed. This specific condition is likely to lead to overestimating the presence and the severity of psychological symptoms. Lastly, many possible mediating variables that existed were not assessed and could not be controlled for, such as specific job demands, number of shifts per month, and redeployment to a new working area.

## 5. Conclusions

Overall, this study shows that HCWs during the COVID-19 outbreak in Italy experienced a high burden of psychological distress. Working in the front-line directly with infected patients, being a part of the MHS, having low personal resources, as well as experiencing higher levels of post-traumatic symptomatology are associated with the risk of burnout. Our findings strongly suggest the need for healthcare organizations to establish psychological support services for providing adequate professional care for HCWs, as well as creating safer work environments to decrease the impact of the COVID-19 epidemic. Societal costs might be higher than expected and go beyond the personal health of HCWs because HCWs’ individual stress is likely to impact the patients’ health both directly and indirectly [57,80]. This is also supported by a recent systematic review on HCWs’ well-being, burnout, and patient safety, showing that poor well-being and moderate to high levels of burnout are related to poor patient safety outcomes such as more medical errors [35]. In conclusion, HCWs need adequate health protection and working conditions as well as recovery programs aiming at empowering resilience and psychological well-being [81] in order to cope better with exceptional times such as the COVID-19 epidemic. This is a call for interventions to psychologically support these professionals in their job, to prevent abandonment, professional errors, personal deterioration, and patients’ dissatisfaction [82,83]. Our results may be of interest also to healthcare stakeholders as healthcare cost savings have been shown by implementing psychological services and interventions within healthcare systems [84,85].

## Figures and Tables

**Table 1:** Sociodemographic, workload characteristics, and psychological symptoms in the total sample and healthcare professions subgroups.

Variables, *N* (%)	Total Sample	Physicians	Nurses	Technicians	UAP ^†^	Hospital Staff	χ^2^	*p*	η^2^
**Overall**	933 (100)	224 (24)	395 (42.3)	99 (10.6)	165 (17.7)	50 (5.4)	-	-	-
Sex									
Men	219 (23.4)	84 (37.5)	70 (17.7)	19 (19.2)	31 (18.8)	14 (28)	35.51	<0.001	0.19
Women	714 (76.5)	140 (62.5)	325 (82.3)	80 (80.8)	134 (81.2)	36 (72)			
**Working position**									
Front-line	709 (76)	180 (80.4)	307 (77.7)	84 (84.8)	111 (67.3)	27 (46)	27.37	<0.001	0.17
Second-line	224 (24)	44 (19.6)	88 (22.3)	15 (15.2)	54 (32.7)	23 (54)			
**Patients’ death**									
Yes	379 (40.6)	92 (41.4)	172 (43.5)	44 (44.4)	66 (40)	5 (10)	21.48	<0.001	0.15
No	554 (59.4)	132 (58.9)	223 (56.5)	55 (55.6)	99 (60)	45 (90)			
**PHQ-9 ^¶^**									
Positives ^§^	453 (57.9)	105 (54.4)	200 (59.3)	52 (62.7)	77 (59.7)	19 (46.3)	4.44	0.35	0.07
**GAD-7 ^¶^**									
Positives ^§^	525 (65.2)	124 (66.6)	232 (67.2)	57 (66.3)	88 (65.2)	24 (54.5)	3.10	0.54	0.06
**IES-R ^¶^**									
Positives ^§^	482 (55)	109 (50.5)	223 (61.1)	50 (53.8)	83 (52.9)	17 (37)	13.67	0.008	0.12
**PFI-tot ^¶^**									
Positives ^§^	239 (25.61)	56 (25)	115 (29.1)	28 (28.3)	31 (18.8)	9 (18)	8.51	0.07	0.09

Notes: ^†^ unlicensed assistive personnel; ^§^ positives: scoring higher than the following cut-off scores = PHQ-9 ≥ 5, GAD-7 ≥ 5, IES-R ≥ 33, PFI-TOT ≥1.33; ^¶^ PHQ-9 = patient health questionnaire; GAD-7 = generalized anxiety disorder scale; IES-R = impact of event scale, revised; PFI-tot = professional fulfillment index total score.

**Table 2:** Psychological symptoms, sociodemographic and occupational characteristics in the total sample and burnout subgroups.

Variables	Total Sample	Burnout (PFI-tot ≥ 1.33)	No-Burnout (PFI-tot < 1.33)	t/χ^2^	*p*	d/η^2^
**Overall, n (%)**	933 (100)	239 (25.61)	694 (74.39)	-	-	-
**Age (years), mean (SD)**	41.77 (12.08)	40.02 (11.01)	42.37 (12.38)	2.60	0.009	0.20
**Sex, n (%)**						
Men	219 (23.4)	51 (21.30)	167 (24.10)	0.73	0.39	0.02
Women	714 (76.5)	188 (78.70)	527 (75.90)			
**Healthcare staff, n (%)**						
Medical	619 (66.3)	171 (71.50)	448 (64.60)	3.89	0.04	0.06
Non-medical	314 (33.7)	68 (28.50)	246 (35.40)			
**Working position, n (%)**						
Front-line	709 (76)	204 (85.40)	505 (72.80)	15.44	<0.001	0.12
Second-line	224 (24)	35 (14.60)	189 (27.20)			
**Patients’ death, n (%)**						
Yes	379 (40.6)	125 (52.30)	254 (36.60)	18.17	<0.001	0.14
No	554 (59.4)	114 (47.70)	440 (63.40)			
**PHQ-9 ^†^, mean (SD)**	6.71 (5.66)	9.85 (5.94)	5.65 (5.15)	9.53	<0.001	0.78
**GAD-7 ^†^, mean (SD)**	7.55 (5.65)	10.74 (5.72)	6.47 (5.21)	9.86	<0.001	0.80
**IES-R ^†^, mean (SD)**	4.92 (2.18)	6.46 (1.95)	4.40 (2.01)	13.28	<0.001	1.03
IES-IT ^†^	1.85 (0.88)	2.35 (0.79)	1.68 (0.84)	10.45	<0.001	0.81
IES-H ^†^	1.65 (0.85)	2.25 (0.79)	1.45 (0.78)	12.96	<0.001	1.02
IES-A ^†^	1.42 (0.69)	1.86 (0.66)	1.27 (0.63)	11.96	<0.001	0.93
**PWB-R ^†^, mean (SD)**	90.97 (12.33)	86.15 (13.60)	92.61 (11.43)	6.68	<0.001	0.54
PWB-A ^†^	15.94 (3.18)	15.57 (3.51)	16.07 (3.06)	1.93	0.05	0.16
PWB-EM ^†^	14.85 (3.23)	13.69 (3.64)	15.24 (2.99)	6.07	<0.001	0.49
PWB-PG ^†^	16.83 (2.85)	15.83 (2.99)	17.17 (2.73)	5.97	<0.001	0.48
PWB-PR ^†^	15.66 (3.47)	14.60 (3.61)	16.02 (3.35)	5.15	<0.001	0.42
PWB-PL ^†^	15.46 (3.03)	14.85 (3.27)	15.67 (2.92)	3.40	<0.001	0.26
PWB-SA ^†^	14.72 (3.59)	13.59 (3.86)	15.11 (3.42)	5.31	<0.001	0.43

Notes: ^†^ PFI-tot = professional fulfillment index total score; PF = professional fulfillment; WE = working exhaustion; ID = interpersonal disengagement; PHQ-9 = patient health questionnaire; GAD-7 = generalized anxiety disorder scale; IES-R = impact of event scale, revised; IES-IT = intrusive thoughts; IES-H = hyperarousal; IES-A = avoidance; PWB-R = psychological well-being scales, revised; PWB-A = autonomy; PWB-EM = environmental mastery; PWB-PG = personal growth; PWB-PR = positive relations with others; PWB-PL = purpose in life; PWB-SA = self-acceptance.

**Table 3:** Pearson’s correlation between burnout subscales and total score, and study variables.

Variables	PFI-tot ^‡^	PFI-PF ^‡^	PFI-WE ^‡^	PFI-ID ^‡^
**Sex ^†^**	−0.07 *	0.06 *	−0.16 **	0.04
**MHS ^†^**	0.09 **	−0.16 **	0.07 *	0.08 *
**Front-line ^†^**	−0.01	−0.07 *	−0.04	0.02
**Patients’ death^†^**	0.19 **	−0.04	0.24 **	0.02
**Age**	−0.13 **	0.05	−0.14 **	−0.08 *
**PHQ-9 ^‡^**	0.47 **	−0.34 **	0.52 **	0.27 **
**GAD-7 ^‡^**	0.50 **	−0.33 **	0.58 **	0.25 **
**IES-R ^‡^**	0.57 **	−0.30 **	0.63 **	0.32 **
IES-IT ^‡^	0.48**	−0.27 **	0.45 **	0.35 **
IES-H ^‡^	0.49 **	−0.23 **	0.59 **	0.23 **
IES-A ^‡^	0.56 **	−0.32 **	0.62 **	0.32 **
**PWB-R ^‡^**	−0.31 **	0.33 **	−0.22 **	−0.29 **
PWB-A ^‡^	−0.12 **	0.14 **	−0.05	−0.15 **
PWB-EM ^‡^	−0.27 **	0.30 **	−0.26 **	−0.19 **
PWB-PG ^‡^	−0.27 **	0.31 **	−0.19 **	−0.26 **
PWB-PR^‡^	−0.23 **	0.22 **	−0.12 **	−0.27 **
PWB-PL ^‡^	−0.14 **	0.20 **	−0.08 *	−0.15 **
PWB-SA ^‡^	−0.26 **	0.30 **	−0.22 **	−0.21 **

Notes: * *p* < 0.05; ** *p* < 0.01; ^†^ point-biserial correlation coefficient; **^‡^** PFI-tot = professional fulfillment index total score; PF = professional fulfillment; WE = working exhaustion; ID = interpersonal disengagement; MHS = medical healthcare staff; PHQ-9 = patient health questionnaire; GAD-7 = generalized anxiety disorder scale; IES-R = impact of event scale, revised; IES-IT = intrusive thoughts; IES-H = hyperarousal; IES-A = avoidance; PWB-R = psychological well-being scales, revised; PWB-A = autonomy; PWB-EM = environmental mastery; PWB-PG = personal growth; PWB-PR = positive relations with others; PWB-PL = purpose in life; PWB-SA = self-acceptance.

**Table 4:** Hierarchical regression analysis with burnout total score as a dependent variable.

Variables	*B*	*SE*	β	ΔR²	R	R²
**Step 1**				0.02	0.16	0.02
Sex	0.09	0.05	0.05			
Age	0.00	0.00	−0.03			
**Step 2**				0.04	0.26	0.06
+ Patients’ death	0.08	0.05	0.05			
+ Working position	0.11	0.05	0.07 *			
+ Healthcare staff	0.09	0.04	0.06 *			
**Step 3**				0.09	0.39	0.16
+ PWB-R ^†^	−0.01	0.00	−0.14 ***			
**Step 4**				0.22	0.61	0.38
+ PHQ-9 ^†^	0.00	0.01	0.03			
+ GAD-7 ^†^	0.01	0.01	0.09			
+ IES-R ^†^	0.14	0.01	0.43 ***			

Notes: Table 4 reflect betas after step 4. * *p* < 0.05; *** *p* < 0.001. ^†^ PWB-R = psychological well-being scales, revised; PHQ-9 = patient health questionnaire; GAD-7 = generalized anxiety disorder scale; IES-R = impact of event scale, revised.

## Data Availability

The data presented in this study are available on request from the corresponding author. The data are not publicly available due to privacy restrictions.

## References

[1] World Health Organization Coronavirus Disease 2019 (COVID-19): Situation Report—79. https://www.who.int/docs/default-source/coronaviruse/situation-reports/20200408-sitrep-79-covid-19.pdf?sfvrsn=4796b143_6.

[2] Luo M., Guo L., Yu M., Wang H. (2020). The Psychological and Mental Impact of Coronavirus Disease 2019 (COVID-19) on Medical Staff and General Public—A Systematic Review and Meta-analysis. Psychiatry Res..

[3] Italian Health Ministry. (n.d.). New Coronavirus Update. http://www.salute.gov.it/nuovocoronavirus.

[4] National Institute of Health. COVID-19 Epidemic. National Upgrade February 10. https://www.epicentro.iss.it/coronavirus/bollettino/Bollettino-sorveglianza-integrata-COVID-19_10-febbraio-2021.pdf.

[5] Chen Q., Liang M., Li Y., Guo J., Fei D., Wang L., He L., Sheng C., Cai Y., Li X. (2020). Mental health care for medical staff in China during the COVID-19 outbreak. Lancet Psychiatry.

[6] Rossi R., Socci V., Pacitti F., Di Lorenzo G., Di Marco A., Siracusano A., Rossi A. (2020). Mental Health Outcomes Among Frontline and Second-Line Health Care Workers During the Coronavirus Disease 2019 (COVID-19) Pandemic in Italy. JAMA Netw. Open.

[7] Xiang Y.T., Yang Y., Li W., Zhang L., Zhang Q., Cheung T., Ng C.H. (2020). Timely mental health care for the 2019 novel coronavirus outbreak is urgently needed. Lancet Psychiatry.

[8] Lu W., Wang H., Lin Y., Li L. (2020). Psychological status of medical workforce during the COVID-19 pandemic: A cross-sectional study. Psychiatry Res..

[9] Xing J., Sun N., Xu J., Geng S., Li Y. (2020). Study of the mental health status of medical personnel dealing with new coronavirus pneumonia. PLoS ONE.

[10] Zhang W.R., Wang K., Yin L., Zhao W.F., Xue Q., Peng M., Min B.Q., Tian Q., Leng H.X., Du J.L. (2020). Mental Health and Psychosocial Problems of Medical Health Workers during the COVID-19 Epidemic in China. Psychother. Psychosom..

[11] Conti C., Fontanesi L., Lanzara R., Rosa I., Porcelli P. (2020). Fragile heroes. The psychological impact of the COVID-19 pandemic on health-care workers in Italy. PLoS ONE.

[12] Muller R.A.E., Stensland R.S.Ø., van de Velde R.S. (2020). The mental health impact of the covid-19 pandemic on healthcare workers, and interventions to help them: A rapid systematic review. Psychiatry Res..

[13] Maslach C., Jackson S.E. (1981). The measurement of experienced burnout. J. Organ. Behav..

[14] Honkonen T., Ahola K., Pertovaara M., Isometsä E., Kalimo R., Nykyri E., Aromaa A., Lönnqvist J. (2006). The association between burnout and physical illness in the general population: Results from the Finnish Health 2000 Study. J. Psychosom. Res..

[15] Shirom A., Toker S., Melamed S., Berliner S., Shapira I. (2013). Burnout and vigor as predictors of the incidence of hyperlipidemia among healthy employees. Appl. Psychol. Health Well Being.

[16] Pérez-Francisco D.H., Duarte-Clíments G., del Rosario-Melián J.M., Gómez-Salgado J., Romero-Martín M., Sánchez-Gómez M.B. (2020). Influence of workload on primary care nurses’ health and burnout, patients’ safety, and quality of care: Integrative review. Healthcare.

[17] Stehman C.R., Testo Z., Gershaw R.S., Kellogg A.R. (2019). Burnout, Drop Out, Suicide: Physician Loss in Emergency Medicine, Part I. West J. Emerg. Med..

[18] Amoafo E., Hanbali N., Patel A., Singh P. (2015). What are the significant factors associated with burnout in doctors?. Occup. Med..

[19] Molero Jurado M., Pérez-Fuentes M., Gázquez Linares J., Simón Márquez M., Martos Martínez Á. (2018). Burnout Risk and Protection Factors in Certified Nursing Aides. Int. J. Environ. Res. Public Health.

[20] Cavanaugh K.J., Lee H.Y., Daum D., Chang S., Izzo J.G., Kowalski A., Holladay C.L. (2020). An Examination of Burnout Predictors: Understanding the Influence of Job Attitudes and Environment. Healthcare.

[21] West C.P., Dyrbye L.N., Erwin P.J., Shanafelt T.D. (2016). Interventions to prevent and reduce physician burnout: A systematic review and meta-analysis. Lancet.

[22] Grassi L., Magnani K. (2000). Psychiatric morbidity and burnout in the medical profession: An Italian study of general practitioners and hospital physicians. Psychother. Psychosom..

[23] Messerotti A., Banchelli F., Ferrari S., Barbieri E., Bettelli F., Bandieri E., Giusti D., Catellani H., Borelli E., Colaci E. (2020). Investigating the association between physicians self-efficacy regarding communication skills and risk of “burnout”. Health Qual. Life Outcomes.

[24] Morgantini L.A., Naha U., Wang H., Francavilla S., Acar O., Flores J.M., Crivellaro S., Moreira D., Abern M., Eklund M. (2020). Factors Contributing to Healthcare Professional Burnout during the COVID-19 Pandemic: A Rapid Turnaround Global Survey. medRxiv.

[25] Barello S., Palamenghi L., Graffigna G. (2020). Burnout and somatic symptoms among frontline healthcare professionals at the peak of the Italian COVID-19 pandemic. Psychiatry Res..

[26] Amanullah S., Ramesh Shankar R. (2020). The Impact of COVID-19 on Physician Burnout Globally: A Review. Healthcare.

[27] Liu Y., Prati L.M., Perrewe P.L., Ferris G.R. (2008). The relationship between emotional resources and emotional labor: An exploratory study. J. Appl. Soc. Psychol..

[28] Ryff C.D. (1989). Happiness is everything, or is it? Explorations on the meaning of psychological well-being. J. Pers. Soc. Psychol..

[29] Ryff C.D., Singer B.H., Love G.D. (2004). Positive health: Connecting well-being with biology. Philos. Trans. R. Soc. Lond. B Biol. Sci..

[30] Ryff C.D., Dienberg Love G., Urry H.L., Muller D., Rosenkranz M.A., Friedman E.M., Davidson R.J., Singer B. (2006). Psychological well-being and ill-being: Do they have distinct or mirrored biological correlates?. Psychother. Psychosom..

[31] Chida Y., Steptoe A. (2008). Positive psychological well-being and mortality: A quantitative review of prospective observational studies. Psychosom. Med..

[32] Hamilton N.A., Nelson C.A., Stevens N., Kitzman H. (2007). Sleep and psychological well-being. Soc. Indic. Res..

[33] Keyes C.L.M. (2005). Chronic physical conditions and aging: Is mental health a potential protective factor?. Ageing Int..

[34] Keyes C.L.M., Grzywacz J.G. (2005). Health as a complete state: The added value in work performance and healthcare costs. J. Occup. Environ. Med..

[35] Hall L.H., Johnson J., Watt I., Tsipa A., O’Connor D.B. (2016). Healthcare Staff Wellbeing, Burnout, and Patient Safety: A Systematic Review. PLoS ONE.

[36] Kalimo R., Pahkin K., Mutanen P., Topipinen-Tanner S. (2003). Staying well or burning out at work: Work characteristics and personal resources as long-term predictors. Work Stress.

[37] Mäkikangas A., Kinnunen U. (2003). Psychosocial work stressors and well-being: Self-esteem and optimism as moderators in a one-year longitudinal sample. Pers. Individ. Differ..

[38] Tremblay M.A., Messervey D. (2011). The Job Demands-Resources model: Further evidence for the buffering effect of personal resources. SA J. Ind. Psychol..

[39] Van den Broeck A., Van Ruysseveldt J., Smulders P., De Witte H. (2011). Does an intrinsic work value orientation strengthen the impact of job resources? A perspective from the Job Demands–Resources Model. Eur. J. Work Organ. Psychol..

[40] Italian Health Ministry. The Staff of the ASL and Public and Equivalent Hospitalization Institutes. Year 2013. http://www.salute.gov.it/imgs/C_17_pubblicazioni_2870_allegato.pdf.

[41] World Medical Association (2013). World Medical Association Declaration of Helsinki Ethical Principles for Medical Research Involving Human Subjects. J. Am. Med. Ass..

[42] Trockel M., Bohman B., Lesure E., Hamidi M.S., Welle D., Roberts L., Shanafelt T. (2018). A brief instrument to assess both burnout and professional fulfillment in physicians: Reliability and validity, including correlation with self-reported medical errors, in a sample of resident and practicing physicians. Acad. Psychiatry.

[43] Kroenke K., Spitzer R.L., Williams J.B. (2001). The PHQ-9: Validity of a brief depression severity measure. J. Gen. Intern. Med..

[44] Levis B., Benedetti A., Thombs B.D. (2019). Accuracy of Patient Health Questionnaire-9 (PHQ-9) for screening to detect major depression: Individual participant data meta-analysis. BMJ.

[45] Spitzer R.L., Kroenke K., Williams J.B., Löwe B. (2006). A brief measure for assessing generalized anxiety disorder: The GAD-7. Arch. Intern. Med..

[46] Löwe B., Decker O., Müller S., Brähler E., Schellberg D., Herzog W., Herzberg P.Y. (2008). Validation and standardization of the Generalized Anxiety Disorder Screener (GAD-7) in the general population. Med. Care.

[47] Kroenke K., Wu J., Yu Z., Bair M.J., Kean J., Stump T., Monahan P.O. (2016). Patient Health Questionnaire Anxiety and Depression Scale: Initial Validation in Three Clinical Trials. Psychosom. Med..

[48] Craparo G., Faraci P., Rotondo G., Gori A. (2013). The Impact of Event Scale—Revised: Psychometric properties of the Italian version in a sample of flood victims. Neuropsychiatr. Dis. Treat..

[49] Weiss D.S., Marmar C.R. (1997). The Impact of Event Scale—Revised, Assessing Psychological Trauma and PTSD.

[50] Tiemensma J., Depaoli S., Winter S.D., Felt J.M., Rus H.M., Arroyo A.C. (2018). The performance of the IES-R for Latinos and non-Latinos: Assessing measurement invariance. PLoS ONE.

[51] Ryff C.D., Keyes C.L.M. (1995). The structure of psychological well-being revisited. J. Pers. Soc. Psychol..

[52] Ruini C., Ottolini F., Rafanelli C., Ryff C., Fava G.A. (2003). Italian validation of Psychological Well-being Scales (PWB). Rivista di Psichiatria.

[53] Cohen J. (1988). Statistical Power Analysis for the Behavioral Sciences.

[54] Lenhard W., Lenhard A. (2016). Calculation of Effect Sizes. Psychometrica.

[55] IBM Corp (2017). Released. IBM SPSS Statistics for Windows, Version 25.0.

[56] De Boer J., Lok A., Van’t Verlaat E., Duivenvoorden H.J., Bakker A.B., Smit B.J. (2011). Work-related critical incidents in hospital-based health care providers and the risk of post-traumatic stress symptoms, anxiety, and depression: A meta-analysis. Soc. Sci. Med..

[57] Privitera M.R., Rosenstein A.H., Plessow F., LoCastro T.M. (2015). Physician burnout and occupational stress: An inconvenient truth with unintended consequences. J. Hosp. Adm..

[58] Del Canale S., Louis D.Z., Maio V., Wang X., Rossi G., Hojat M., Gonnella J.S. (2012). The relationship between physician empathy and disease complications: An empirical study of primary care physicians and their diabetic patients in Parma, Italy. Acad. Med..

[59] Bodenheimer T., Sinsky C. (2014). From triple to quadruple aim: Care of the patient requires care of the provider. Ann. Fam. Med..

[60] Han S., Shanafelt T.D., Sinsky C.A., Awad K.M., Dyrbye L.N., Fiscus L.C., Trockel M., Goh J. (2019). Estimating the Attributable Cost of Physician Burnout in the United States. Ann. Intern. Med..

[61] Chou L.P., Li C.Y., Hu S.C. (2014). Job stress and burnout in hospital employees: Comparisons of different medical professions in a regional hospital in Taiwan. BMJ Open.

[62] Edwards S.T., Marino M., Balasubramanian B.A., Solberg L.I., Valenzuela S., Springer R., Stange K.C., Miller W.L., Kottke T.E., Perry C.K. (2018). Burnout Among Physicians, Advanced Practice Clinicians and Staff in Smaller Primary Care Practices. J. Gen. Intern. Med..

[63] World Health Organization Shortage of Personal Protective Equipment Endangering Health Workers Worldwide. https://www.who.int/news-room/detail/03-03-2020-shortage-of-personal-protective-equipment-endangering-health-workers-worldwide.

[64] Theorell T. (2012). Evaluating life events and chronic stressors in relation to health: Stressors and health in clinical work. Adv. Psychosom. Med..

[65] Kawashima Y., Nishi D., Noguchi H., Usuki M., Yamashita A., Koido Y., Okubo Y., Matsuoka Y.J. (2016). Post-Traumatic Stress Symptoms and Burnout Among Medical Rescue Workers 4 Years After the Great East Japan Earthquake: A Longitudinal Study. Disaster Med. Public Health Prep..

[66] Mattei A., Fiasca F., Mazzei M., Necozione S., Bianchini V. (2017). Stress and Burnout in Health-Care Workers after the 2009 L’Aquila Earthquake: A Cross-Sectional Observational Study. Front. Psychiatry.

[67] Metregiste D., Boucaud-Maitre D., Aubert L., Noubou L., Jehel L. (2020). Explanatory factors of post-traumatic distress and burnout among hospital staff 6 months after Hurricane Irma in Saint-Martin and Saint-Barthelemy. PLoS ONE.

[68] Cieslak R., Shoji K., Douglas A., Melville E., Luszczynska A., Benight C.C. (2014). A meta-analysis of the relationship between job burnout and secondary traumatic stress among workers with indirect exposure to trauma. Psychol. Serv..

[69] Li Z., Ge J., Yang M., Feng J., Qiao M., Jiang R., Bi J., Zhan G., Xu X., Wang L. (2020). Vicarious traumatization in the general public, members, and non-members of medical teams aiding in COVID-19 control. Brain Behav. Immun..

[70] Beck C.T. (2011). Secondary traumatic stress in nurses: A systematic review. Arch. Psychiatr. Nurs..

[71] Hickling E.J., Gibbons S., Barnett S.D., Watts D. (2011). The psychological impact of deployment on OEF/OIF healthcare providers. J. Trauma. Stress.

[72] Hobfoll S.E., Folkman S. (2011). Conservation of resources theory: Its implications for stress, health, and resilience. The Oxford Handbook of Stress, Health, and Coping.

[73] Hobfoll S.E. (2012). Conservation of resources and disaster in cultural context: The caravans and passageways for resources. Psychiatry.

[74] Ruini C., Fava G.A. (2012). Role of well-being therapy in achieving a balanced and individualized path to optimal functioning. Clin. Psychol. Psychother..

[75] Mistretta E.G., Davis M.C., Temkit M.H., Lorenz C., Darby B., Stonnington C.M. (2018). Resilience training for work-related stress among health care workers: Results of a randomized clinical trial comparing in-person and smartphone-delivered interventions. J. Occup. Environ. Med..

[76] Werneburg B.L., Jenkins S.M., Friend J.L., Berkland B.E., Clark M.M., Rosedahl J.K., Preston H.R., Daniels D.C., Riley B.A., Olsen K.D. (2018). Improving Resiliency in Healthcare Employees. Am. J. Health Behav..

[77] Gaiaschi C. (2019). Same job, different rewards: The gender pay gap among physicians in Italy. Gend. Work Organ..

[78] Ramsey S.R., Thompson K.L., McKenzie M., Rosenbaum A. (2016). Psychological research in the internet age: The quality of web-based data. Comput. Hum. Behav..

[79] Rutherford C., Costa D., Mercieca-Bebber R., Rice H., Gabb L., King M. (2016). Mode of administration does not cause bias in patient-reported outcome results: A meta-analysis. Qual. Life Res..

[80] Sonnentag S. (2015). Wellbeing and burnout in the workplace: Organizational causes and consequences. Int. Encycl. Soc. Behav. Sci..

[81] Fava G.A., Cosci F., Sonino N. (2017). Current Psychosomatic Practice. Psychother. Psychosom..

[82] Suñer-Soler R., Grau-Martín A., Flichtentrei D., Prats M., Braga F., Font-Mayolas S., Gras M.E. (2014). The consequences of burnout syndrome among healthcare professionals in Spain and Spanish speaking Latin American countries. Burn. Res..

[83] Patel R.S., Bachu R., Adikey A., Malik M., Shah M. (2018). Factors Related to Physician Burnout and Its Consequences: A Review. Behav. Sci..

[84] Gulliksson M., Burell G., Vessby B., Lundin L., Toss H., Svärdsudd K. (2011). Randomized controlled trial of cognitive behavioral therapy vs standard treatment to prevent recurrent cardiovascular events in patients with coronary heart disease: Secondary Prevention in Uppsala Primary Health Care project (SUPRIM). Arch. Intern. Med..

[85] Layard R., Clark D.M. (2015). Why More Psychological Therapy Would Cost Nothing. Front. Psychol..

